# Venous thromboembolism in women: new challenges for an old disease

**DOI:** 10.1590/1677-5449.200181

**Published:** 2021-06-25

**Authors:** Alvin Oliver Payus, Constance Liew Sat Lin, Azliza Ibrahim, Norlaila Mustafa

**Affiliations:** 1 Universiti Malaysia Sabah – UMS, Kota Kinabalu, Sabah, Malaysia.; 2 Hospital Pengajar Universiti Putra Malaysia – HPUPM, Fakulti Perubatan dan Sains Kesihatan, Serdang, Selangor, Malaysia.; 3 Universiti Kebangsaan Malaysia Medical Centre – UKMMC, Cheras, Kuala Lumpur, Malaysia.

Dear Editor,

We read with great interest the article by Oliveira AL et al., entitled “Venous Thromboembolism in Women: New Challenges for An Old Disease,” which was recently published in volume 19, July 2020.^1^ Firstly, we would like to praise the authors for their effort in summarizing the associations between venous thromboembolism (VTE) and clinical situations peculiar to women in the comprehensive non-systematic review. The authors have written the review in great detail and have outlined an exhaustive list of possible links between VTE and woman in various clinical condition, especially during pregnancy or while taking hormonal treatment such as the oral contraceptive pill and hormonal replacement therapy. However, as readers, we believe it would be of great benefit if additional information could be included, such as the treatment of established pulmonary embolism in pregnant women and a recommended algorithm for diagnosing VTE in pregnant women. Apart from that, in addition to the risk factors mentioned in the review, which include smoking, obesity, and positive family history of VTE, we believe that previous history of trauma, especially to the chest, is also a significant risk factor^2^ which can lead to pulmonary embolism that arises de novo^3^ and warrants inclusion in the review. Nevertheless, we agree with the authors that VTE in women is a major clinical challenge in modern medical practice and believe that this comprehensive review is beneficial and can be used for future reference, especially in implementing guidelines and protocols to improve the quality of life of women needing care.
