# Supplementary material for: Venous thromboembolism in women: new challenges for an old disease
Source: J Vasc Bras. 2021 Jun 25;20:e20200181. doi: 10.1590/1677-5449.200181 (PMC8276646; doi:10.1590/1677-5449.200181)
Supplement: Related texts

# Reply: RESPONSE LETTER

**Authors:** Marcos Arêas Marques

**Affiliations:** 1 Universidade do Estado do Rio de Janeiro – UERJ, Unidade Docente, Assistencial de Angiologia, Rio de Janeiro, RJ, Brasil

## Dear Editor,

We would like to thank you for the opportunity to respond to the issues raised in the letter from Dr Payus and colleagues and to clarify aspects of our article in relation to those concerns. We completely agree that it would have been of great benefit to include information about treatment of established pulmonary embolism in pregnant women and a recommended algorithm for diagnosing VTE in pregnant women, because of the use of limited doses of radiation and the anatomical difficulties in pregnant patients. However, such an article would be very long. Pulmonary embolism after trauma or even after insect bites in pregnant patients is a very interesting scenario, constituting a local response to injury or inflammation. However, further research is warranted to fully understand this pathophysiology. We will be glad to reinforce these points in future articles. Finally, our special thanks go to the Dr. Payus and colleagues for their interest in our paper and for taking the time to express their concerns.
